# Determination of sweetener specificity of horse gut-expressed sweet taste receptor T1R2-T1R3 and its significance for energy provision and hydration

**DOI:** 10.3389/fvets.2024.1325135

**Published:** 2024-02-12

**Authors:** Liberty Smith, Andrew W. Moran, Miran Al-Rammahi, Kristian Daly, Soraya P. Shirazi-Beechey

**Affiliations:** ^1^Institute of Infection, Veterinary and Ecological Sciences, University of Liverpool, Liverpool, United Kingdom; ^2^Department of Physiology, Biochemistry and Pharmacology, College of Veterinary Medicine, University of Al-Qadisiyah, Al-Diwaniyah, Iraq

**Keywords:** horse, taste, nutrition, T1R2-T1R3, dietary additive, low-calorie sweeteners, intestine, oral rehydration

## Abstract

Studies carried out in several species have demonstrated that detection of low-calorie sweeteners in the lumen of the intestine, by the sweet receptor, T1R2-T1R3, initiates a signaling pathway leading to enhanced expression and activity of intestinal Na^+^/glucose cotransporter 1, SGLT1. This results in an increased gut capacity to absorb glucose, sodium chloride and water, the basis for oral rehydration therapy. Horses express T1R2, T1R3 and downstream signaling elements in the intestinal tissue. As such, the potential of sweetener-stimulation of T1R2-T1R3 leading to upregulation of SGLT1 allows the provision of more glucose (energy) and hydration for horses. This is especially important when the need for glucose increases during strenuous exercise, pregnancy, and lactation. There are significant differences among species in the ability to detect sweeteners. Amino acid substitutions and pseudogenization of taste receptor genes underlie these variations. Nothing is known about the sweetener specificity of horse T1R2-T1R3. Using heterologous expression methodology, we demonstrate that sweeteners sucralose, stevia and neohesperidin dihydrochalcone (NHDC) activate horse T1R2-T1R3, but cyclamate does not. Determination of sweetener specificity of equine sweet receptor is crucial for developing suitable dietary additives to optimize glucose absorption, hydration and avoiding the intestinal disease brought about by microbial fermentation of unabsorbed carbohydrate reaching the large intestine.

## 1 Introduction

The sweet taste receptor consisting of heterodimeric G-protein coupled receptor Taste 1 Receptor 2 (T1R2) and Receptor 3 (T1R3) subunits mediates sweet taste perception in humans and other species. The receptor is expressed in taste cells of the lingual epithelium (1, 2). We showed for the first time that the sweet taste receptor and associated G-protein, α-gustducin is expressed in enteroendocrine cells of the intestine in humans and rodents (3, 4). Deletion of either the T1R3 (*Tas1R3*) or α-gustducin (*GNAT3*) genes eliminated the ability of mouse intestine to increase SGLT1 activity and expression in response to dietary glucose or low-calorie sweeteners (4, 5).

Sensing of sugars and sweeteners by the gut expressed T1R2-T1R3 triggers a chemosensing pathway in L-enteroendocrine cells. This results in secretion of the gut hormone glucagon-like peptide 2 (GLP-2). Knocking out the GLP-2 receptor, expressed in enteric neurons, abolishes the ability of mouse intestine to increase SGLT1 expression in response to increased dietary carbohydrate, demonstrating that GLP-2 is part of the sugar-induced pathway in the intestinal mucosa that regulates SGLT1 expression (6, 7). Accordingly, GLP-2 via a neuro-paracrine pathway, enhances activity and expression of SGLT1 in neighboring absorptive enterocytes, leading to an enhanced gut capacity to absorb glucose, sodium chloride, and water (6).

It has been demonstrated that the main route for absorption of glucose in equine small intestine is by SGLT1 (8). Glucose absorbed in the small intestine offers a significant proportion of horse’s energy requirement. This is especially important when the need for glucose increases in conditions such as strenuous exercise, pregnancy, or lactation.

Studies investigating horse gustatory responses to sweet compounds have been mainly performed using an *in vivo* two-bottle preference test, showing that horses display a preference for sucrose, and are able to taste sugar (9). We identified the genes for equine T1R3 and α-gustducin from the available horse genome sequence and cloned T1R2 mRNA from equine lingual epithelium (10). The amino acid sequence deduced from the nucleotide sequence showed significant homology and phylogenetic similarity to T1R2 amino acid sequence to other mammalian species including humans (see Table 1). With the closest homologs being T1R2 of pig and cow (>85% similarity, >75% identity) (10). Table 1 also shows nucleotide and amino acid sequence homology for human and horse T1R3, T1R2, and α-gustducin. We further demonstrated that equine intestine expresses, in L-enteroendocrine cells, the key chemosensing signaling elements T1R2, T1R3, α-gustducin and GLP-2, required for SGLT1 upregulation (10).

**Table 1:** %Homology of human (hu) and horse (hr) sweet receptor components.

Sequence	mRNA identity/%	Protein identity/%	Protein similarity/%
T1R2	83.36	75.33	84.86
T1R3	80.60	73.45	82.57
GNAT3	88.54	96.33	98.59

Alignments were compiled using SnapGene software 7.1 (Dotmatics, Boston, MA). Accession numbers used in the comparisons: hu T1R2, NM_152232.6; hu T1R3, NM_152228; hu GNAT3, NM_001102386.3; hr T1R2, XM_023635318.1; hr T1R3, XM_001915778.4; hr GNAT3, XM_001487934.5.

Sweeteners have been used worldwide as dietary additives in post-weaning animals for increasing glucose absorption, rehydration, and prevention of weaning related diarrhea (6, 11). No studies at the molecular level have been conducted on equine sweet taste preference to date, particularly in response to low-calorie sweeteners.

In this paper, we have assessed the ability of several sweeteners in activating horse T1R2-T1R3 using a heterologous expression system (12). We show here that sucralose, stevia 80 and NHDC, at much lower concentrations than glucose, activate horse T1R2-T1R3, whereas cyclamate does not. Determination of sweetener specificity of equine sweet taste receptor is essential for developing equine-specific dietary additives to optimize glucose and water absorption, providing energy and hydration, and preventing intestinal disorders in hard working horses fed high grain diets.

## 2 Materials and methods

### 2.1 Compounds and reagents

Sodium cyclamate (71440), NHDC (N8757), sucralose (69293), stevia 80 (06295001) and glucose (G8270) were purchases from Sigma Aldrich Co. Ltd., Dorset, United Kingdom. The anti-FLAG (ab1162) antibody was purchased from Abcam PLC, Cams, United Kingdom and the goat anti-HSV (NB600-513) from Bio-Techne Ltd., Abingdon, United Kingdom. The Fluorescein (FITC)-conjugated donkey anti rabbit (711-095-152) and Cy3-conjugated donkey anti goat IgG (705–165-147) were from Jackson ImmunoResearch Laboratories, West Grove, PA, United States. Lipofectamine 2000 (11668019) was from Life Technologies, Paisley, United Kingdom.

### 2.2 Horse sweet taste receptor constructs

Full length coding sequences (CDS) of horse (h) *hTas1r2* and *hTas1r3* genes were synthesized (Eurofins Genomics, Ebersberg, Germany) and cloned into modified expression vectors pcDN3/TO (hTas1r2), and pcDNA5/FRT/PM (hTas1r3). The receptor sequences were flanked with, an amino terminal sst-tag (coding for first 45 amino acid residues of rat somatostatin receptor type 3) to facilitate an efficient translocation of the receptor subunits to the cell membrane (13), and carboxy terminal FLAG- or HSV-tags to enable immunological detection (14).

### 2.3 Immunocytochemistry

Immunocytochemistry was performed as described before (12). Briefly, human embryonic kidney (HEK)-293PEAKrapid cells that stably express the chimeric G-protein subunit, Gα15, were transiently transfected with the horse sweet receptor constructs (Tas1r2pcDNA3/TO and Tas1r3pcDNA5/FRT/PM) or with an empty pcDNA3/TO vector (negative control) using Lipofectamine2000 according to manufacturer’s instruction. T1R2 and T1R3 proteins were observed using rabbit anti-FLAG and goat anti-HSV primary antibodies. Cells were further incubated with fluorescein (FITC)-conjugated donkey anti rabbit or Cy3-conjugated donkey anti goat IgG, and subsequently mounted and visualized. Images were captured with a DS-Fi3 fluorescent camera (Nikon Instruments, United Kingdom) with merging of images carried out using NIS-Elements Basic Research software (*vs* 5.30.04, Nikon Instruments, United Kingdom).

### 2.4 Functional expression of horse T1R2-T1R3

For the assessment of horse T1R2-T1R3 function, HEK293PEAKrapid Gα15 cells, were seeded into poly-D-lysing coated 96-well plates and transfected with either horse sweet receptor constructs or empty vector (as described above under immunocytochemistry). After 48 h incubation, cells were loaded with Ca^2+^-sensitive Fluo-4 AM fluorescent dye as described previously (12). Sweeteners, sucralose, stevia 80, NHDC, cyclamate and the natural sugar, glucose were diluted to desired concentrations. Ca^2+^ responses of transfected cells upon sweetener application were measured using Flex Station3 microplate reader (Molecular Devices UK Ltd., Wokingham, United Kingdom). For each trace, sweetener was added 22 s after the start of scan, with fluorescent measurements (excitation 485 nm; emission 525 nm) taken every 1.4 for 500 s as described (12). Data were calculated from at least three set of experiments. For the calculation of concentration curves, mean signals of wells receiving the same concentration of test compounds were used, with fluorescence changes of corresponding mock-transfected cells subtracted (∆F). Signals were then normalized to background fluorescence (F). Plots of signal amplitude (ΔF/F) against concentration were used to calculate the EC_50_ values as reported before (12). The EC_50_ values of agonist-receptor interaction were calculated by nonlinear regression of amplitude plots to the function f(x) = 100/[1 + (EC_50_/x)nH], where x is the agonist concentration and nH is the Hill coefficient (15). The EC_50_ is a measure of concentration and shows the amount of sweetener needed to elicit the half maximal responses.

## 3 Results

### 3.1 Heterologous expression and functional characterization of horse T1R2-T1R3 response to low-calorie sweeteners

**Figure 1:**
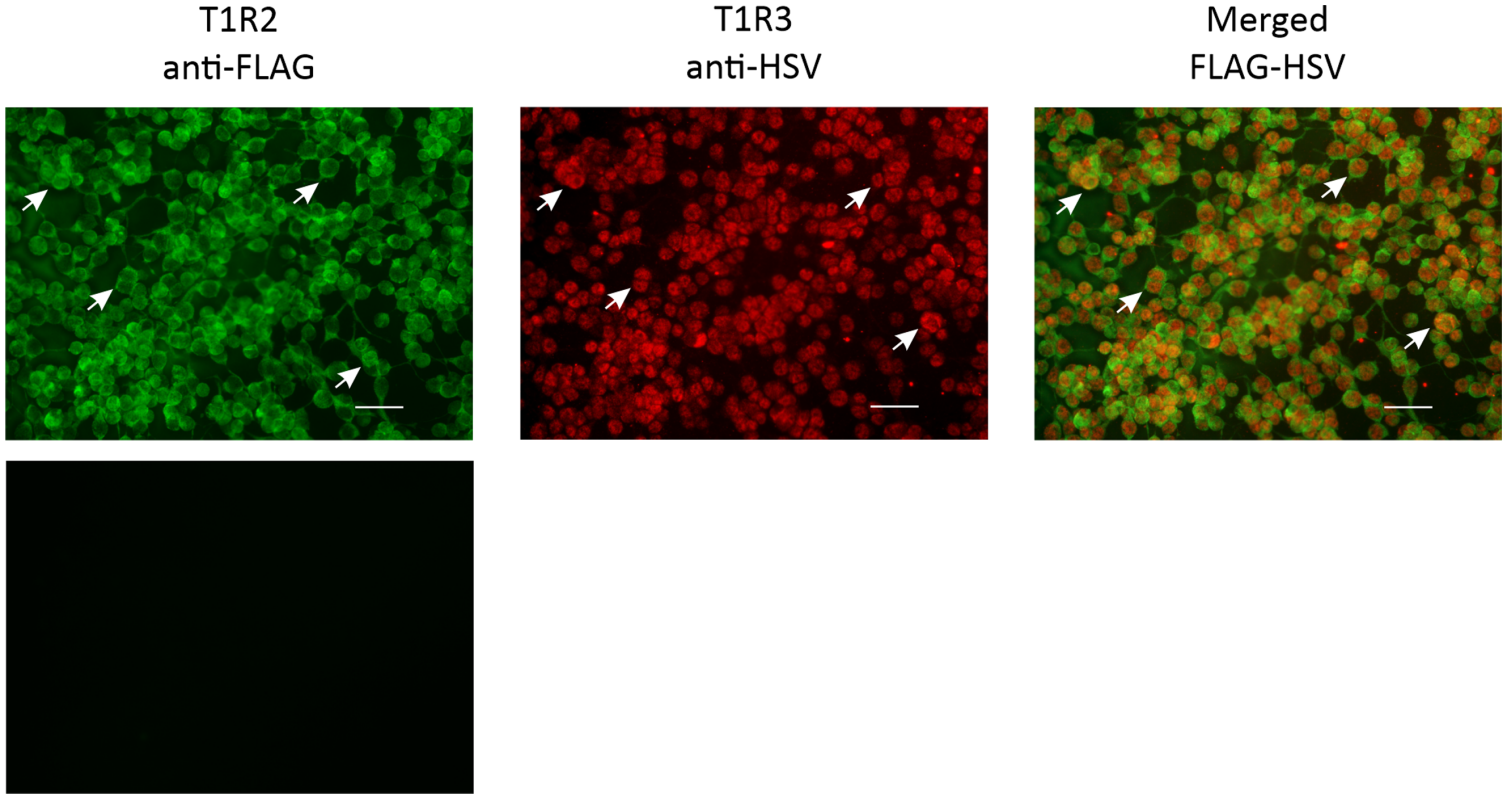
Immunocytochemical detection of transfected equine T1R2-T1R3. HEK293PEAKrapid Gα15 cells were transfected with cDNA coding for horse T1R2 and T1R3 subunits and probed with antibodies directed against the C-terminal tags FLAG and HSV. Arrows point to cells which express T1R2 (green), T1R3 (red) and when overlaid showing co-expression of T1R2 and T1R3 (orange) in the same cell and at the plasma membrane. Cells transfected with empty vector served as a negative control. Images are 100X magnified. Scale bar = 100 μm.

It has been demonstrated, both *in vitro* and *in vivo*, that T1R2 and T1R3 combine to function as the sweet receptor, and that T1R2 or T1R3 alone cannot act as the sweet receptor (1, 16, 17). Furthermore, in various species studied, the receptor resides on the cell membrane of endocrine cells (12, 18). As such the prerequisite for identification of sweeteners that may activate the horse sweet receptor by heterologous expression was that receptor proteins are co-expressed and are present at the cell membrane. We showed by immunofluorescence, that in HEK293PEAKrapid cells transfected either with horse T1R2 or T1R3 receptor construct, T1R2 and T1R3 proteins were expressed, and majority of cells that contained T1R2, also possessed T1R3 receptor subunit; they were also located on the plasma membrane of cells (Figure 1).

To identify the potential of sweetener compounds, sucralose, stevia 80, NHDC, cyclamate (in comparison to glucose) activating horse T1R2-T1R3, we screened HEK293PEAKrapid cells stably expressing the G protein subunit Gα15 and transiently transfected with horse sweet receptor constructs. Association of expressed taste receptor subunits with Gα15 links extracellular activation of the functional sweet taste receptor to elevation of cytosolic Ca^2+^ concentrations (19) and allowing calcium fluorescence to be measured. Mock transfected control cells, when challenged with the same compounds did not show any calcium signals.

**Figure 2:**
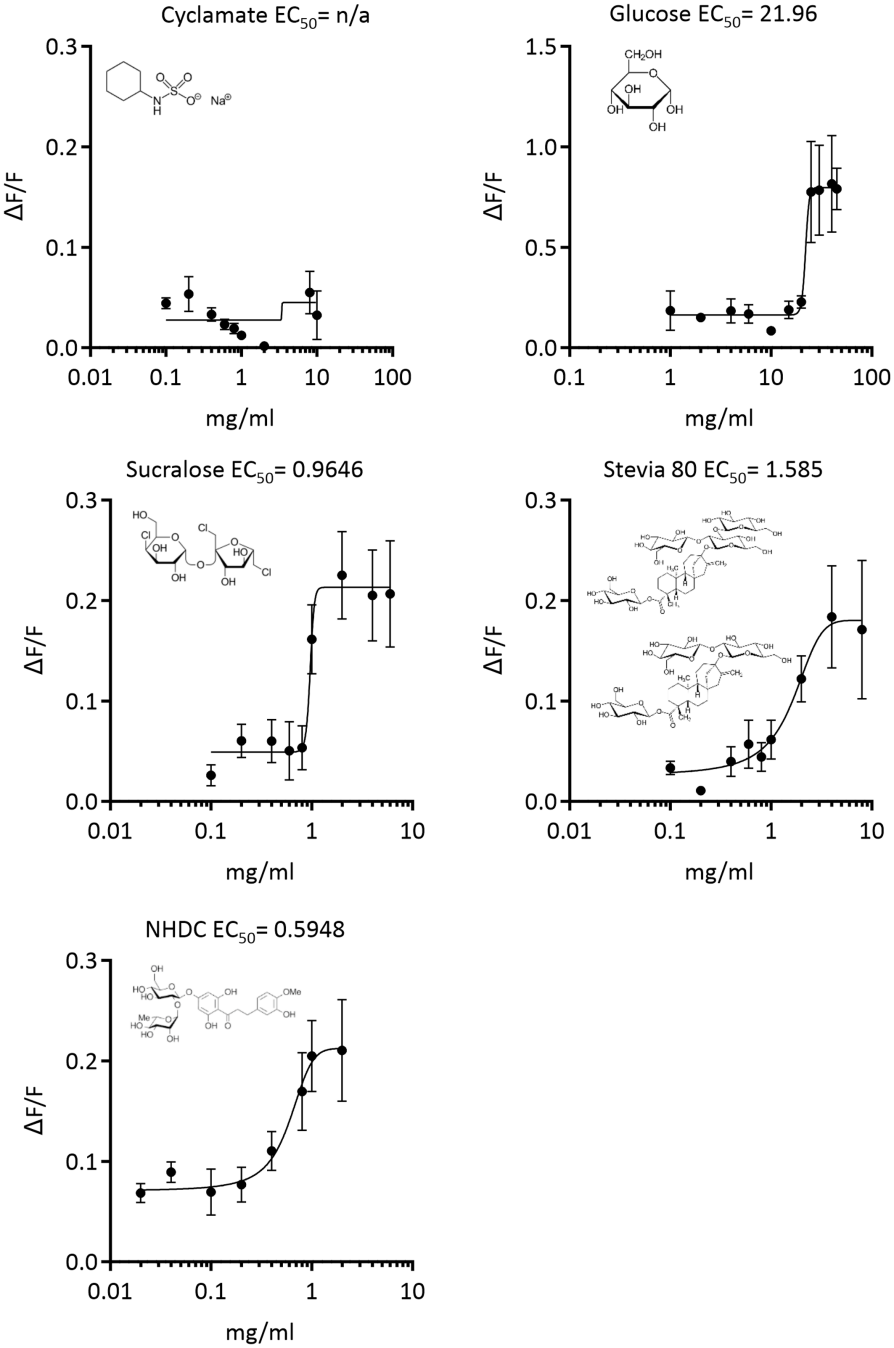
Concentration response curves of equine T1R2-T1R3 activation in response to low-calorie sweeteners, sucralose, stevia, NHDC, cyclamate and the sugar, glucose. Signal amplitude of Ca2+ fluorescence (ΔF/F) vs. concentration (mg/mL) was used to calculate EC50 values of sweetener-receptor interaction by nonlinear regression.

Results demonstrate that sucralose, stevia 80 and NHDC, at much lower concentrations than glucose, activate equine T1R2/T1R3 (Figure 2). No activation of equine T1R2/T1R3 was observed with cyclamate. The potency of sweetener activation, as indicated by EC_50_ value is NHDC (EC_50_ = 0.598 mg/mL) > Sucralose (EC_50_ = 0.965 mg/mL) > Stevia 80 (EC_50_ = 1.585 mg/mL) > glucose (EC_50_ = 22 mg/mL; Figure 2).

The sweeteners used are all known to activate human T1R2-T1R3 (17, 20, 21).

## 4 Discussion

Horse’s natural diet is pasture forage (grass). However, to provide enough energy for the demand of work and performance, domesticated horses are fed diets supplemented with soluble carbohydrates generally in the form of grain (starch). Starch is hydrolyzed in the small intestine to D-glucose, which is transported into the absorptive enterocytes via SGLT1. SGLT1 upregulation leading to increased glucose absorption will provide more energy (glucose) for the horse. It also prevents microbial fermentation of unabsorbed glucose reaching the large intestine. Microbial fermentation of carbohydrates entering the horse’s hind gut leads to production of lactic acid and gasses resulting in colic, a major cause of equine mortality (22–24).

Studies carried out in several species have demonstrated that sensing of low-calorie sweeteners in the lumen of the intestine, by the sweet receptor T1R2-T1R3, initiates a signaling pathway leading to enhanced expression of SGLT1 (6, 25–27). As such, the potential of sweetener-stimulation of horse T1R2-T1R3 leading to upregulation of SGLT1, can increase glucose absorption. The Horse intestine expresses the sweet taste receptor, T1R2-T1R3, the associated G-protein α-gustducin and the carbohydrate responsive gut hormone GLP-2, required for sweet sensing and upregulation of intestinal glucose transport (10), and in this study we show that horse T1R2-T1R3, is activated by a number of sweeteners.

No studies at the molecular level have been conducted on equine sweet taste preference to date, particularly in response to low-calorie sweeteners. Stevia extracts have been used as a sweetener for horses (28), however it has not been shown if stevia stimulates horse sweet taste receptor. We show here, that sweeteners NHDC, sucralose and Stevia 80 at much lower concentrations than glucose activate equine T1R2-T1R3. In contrast, cyclamate does not activate the receptor.

**Figure 3:**
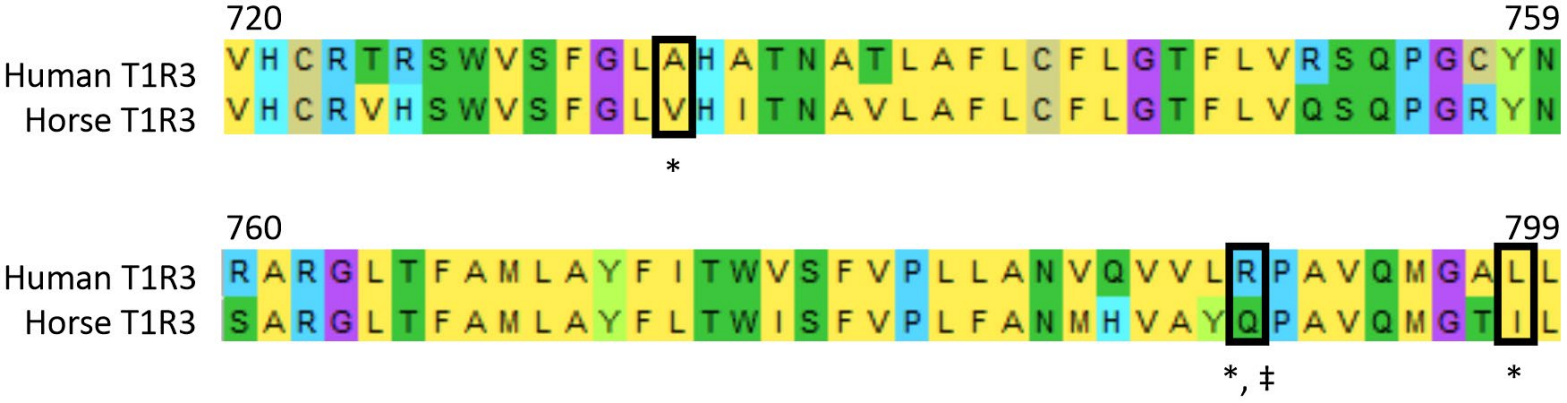
Alignment of T1R3 amino acid sequences of human and horse. Alignment was determined using Mega (version 10.0.5). Black boxes show residues critical for human specific cyclamate activation (‡) and human specific lactisole inhibition (*). These residues are mutated in horse T1R3 sequence. Amino acid Numbers refer to residues in the human T1R3 amino acid sequence.

The inability of horse T1R2-T1R3 to sense cyclamate is not confined to the horse. Mouse T1R2-T1R3 is not also activated by this sweetener due to an amino acid substitution, as R790 in transmembrane domain of human T1R3 is substituted to Q795 in mouse T1R3 (29). Moreover, mutation of R790 in human T1R3 abolishes the ability of human T1R2-T1R3 to be stimulated by cyclamate (29) demonstrating the key role played by this amino acid in cyclamate binding to human T1R3. Sequence analysis reveals that R790 is also substituted in horse to Q789 (see Figure 3) and the likely reason for cyclamate’s inability to activate horse sweet taste receptor.

Lactisole (propionic acid, 2-[4-methoxyphenol] salt) also interacts with transmembrane domain of human T1R3 to inhibit human sweet taste (30). It has been proposed that lactisole inhibition of horse gut-expressed sweet taste receptor may have the potential for the inhibition of intestinal glucose uptake and can be used as a therapeutic strategy for the management of insulin dysregulation in horses with metabolic syndrome (31). However, amino acid residues critical for lactisole inhibition of human T1R3 A733, R790 and L798 (30) are mutated in horse T1R3 to V732 Q789 and I798, respectively (Figure 3). This results in failure of lactisole to inhibit horse sweet receptor and by extension intestinal glucose uptake, invalidating its use as the inhibitor of horse T1R2-T1R3.

The strategy presented in this paper provides novel insights into properties of horse T1R2-T1R3 in response to sweeteners. A close correlation of *in vitro* T1R2-T1R3 stimulation with sweetener-induced activation of gut-expressed T1R2-T1R3 in other species (12), provides support that a similar *in vitro* and *in vivo* association may exist in the horse.

The results presented in this paper provide the baseline knowledge for the development of new effective and targeted products. This will allow a scientifically-evaluated basis for feed formulations for increasing energy provision, improving hydration and reducing intestinal dysfunction in hard working horses given high grain diets.

## Data availability statement

The raw data supporting the conclusions of this article will be made available by the authors, without undue reservation.

## Ethics statement

Ethical approval was not required for the studies on humans in accordance with the local legislation and institutional requirements because only commercially available established cell lines were used. Ethical approval was not required for the studies on animals in accordance with the local legislation and institutional requirements because only commercially available established cell lines were used.

## Author contributions

LS: Data curation, Investigation, Methodology, Writing – original draft. AM: Formal analysis, Supervision, Writing – original draft. MA-R: Data curation, Methodology, Supervision, Writing – original draft. KD: Methodology, Writing – original draft. SS-B: Conceptualization, Funding acquisition, Project administration, Supervision, Writing – original draft, Writing – review & editing.
